# The *ANGPTL*8 rs2278426 (C/T) Polymorphism Is Associated with Prediabetes and Type 2 Diabetes in a Han Chinese Population in Hebei Province

**DOI:** 10.1155/2020/1621239

**Published:** 2020-12-02

**Authors:** Guangsen Hou, Yong Tang, Luping Ren, Yunpeng Guan, Xiaoyu Hou, Guangyao Song

**Affiliations:** ^1^Department of Internal Medicine, Hebei Medical University, Shijiazhuang, Hebei 050017, China; ^2^Endocrinology Department, Hebei General Hospital, Shijiazhuang, Hebei 050051, China

## Abstract

**Background:**

Our aim was to investigate the association between the genetics of the angiopoietin protein-like 8 (*ANGPTL*8) rs2278426 (C/T) polymorphism with prediabetes (pre-DM) and type 2 diabetes (T2DM) in a Han Chinese population in Hebei Province, China.

**Methods:**

We enrolled 1,460 participants into this case-control study: healthy controls, *n* = 524; pre-DM, *n* = 460; and T2DM: *n* = 460. Ligase assays on blood samples from all participants were used to identify polymorphisms. Differences in genotype and allele distributions were compared by the chi-square test and one-way analysis of variance, and a post hoc pairwise analysis was performed using the Bonferroni test. The logistic regression technique was adjusted for age, sex, and body mass index.

**Results:**

The frequency of the TT (10.9%) genotype was significantly higher in pre-DM patients than in controls (odds ratio [OR] = 1.696, 95% confidence interval [CI] = 1.026–2.802, *P*=0.039). In the T2DM group, the CT (48%) and TT (15%) genotypes were significantly higher compared with those in the control group (CT : OR = 1.384, 95% CI = 1.013–1.890, *P*=0.041; TT : OR = 2.530, 95% CI = 1.476–4.334, *P*=0.001). The frequency of the T allele was significantly higher in the pre-DM (32.8%) and T2DM (39%) groups compared with the control group (26.9%) and was significantly associated with an increased risk of pre-DM (OR = 1.253, 95% CI = 1.017–1.544, *P*=0.034) and T2DM (OR = 1.518, 95% CI = 1.214–1.897, *P*=0.001). Furthermore, insulin levels in the pre-DM and T2DM groups were significantly decreased in those with the TT genotype compared with the CC and CT genotypes.

**Conclusion:**

*ANGPTL8* rs2278426 may be involved in the mechanism of insulin secretion and could lead to an increased risk of pre-DM and T2DM.

## 1. Introduction

The high incidence, rising prevalence, and low control rate of type 2 diabetes mellitus (T2DM) have made it one of the most common diseases in the world. In China, 11.6% of adults have been reported to have diabetes, while prediabetes (pre-DM), a precursor to T2DM, was found in 50.1% of the population [1]. The main pathogenesis of diabetes is an absolute or relative reduction in insulin secretion [2–6]. Blood sugar levels that rise continuously during the development of diabetes explain the large numbers of patients with pre-DM; without any intervention, nearly half of these people will develop diabetes within 10 years [7]. Pre-DM is a precondition of T2DM that, if not detected early, can progress to T2DM, leading to delays in diagnosis and treatment. The development of diabetes is influenced by environmental and genetic factors [8–11]. Although there are many candidate genes involved in the development of T2DM, few studies have been conducted in pre-DM populations, and those have showed inconsistent results.

The angiopoietin-like protein 8 (*ANGPTL*8, also known as lipasin or betatrophin) mainly plays important roles in lipid transport and metabolism [12, 13]. *ANGPTL*8, as a member of the angiopoietin-like protein family, is located on open reading frame 80 of chromosome 19, in the corresponding intron of *DOCK*6. *ANGPTL*8 is mainly expressed in the liver in humans [14] and in the liver and white and brown adipose tissue in mice [15]. *ANGPTL*8 was first identified as betatrophin by Melton and colleagues as a hormone that acts as a regulatory factor to significantly promote *β*-cell proliferation in the pancreas [16]. *ANGPTL*8 directly interacts with the insulin-regulated *ANGPTL*3 (the interacting partner of *ANGPTL*8) to regulate the activity of lipoprotein lipase (LPL), a key enzyme in the lipoprotein lipolysis pathway [17]. *ANGPTL*8 is related to two important processes leading to the development of T2DM, insulin resistance, and lipid metabolism and can regulate *β*-cell replication in insulin resistance [18, 19]. Guo et al. [20] found that overexpression of *ANGPTL*8 in the mouse liver resulted in improved blood glucose status in mice, and *ANGPTL*8 in human HepG2 cells may affect glycogen synthesis and gluconeogenesis. Another study found that the targeted expression of *ANGPTL*8 in normal adult rats induced the proliferation of margin cells [21]. Several studies have shown that *ANGPTL*8 in circulating blood is elevated in patients with diabetes [22–24] and obesity [24, 25].

Recently, associations between *ANGPTL*8, diabetes, and blood lipids have aroused a great deal of interest. The rs2278426 (C > T) polymorphism at c194 is very common in *ANGPTL*8 studies and has been shown to affect the activated form of *ANGPTL*3 and can be exposed to LPL inhibition, which is related to low plasma levels of low-density lipoprotein cholesterol (LDL-C) and high-density lipoprotein cholesterol (HDL-C) [17]. Some studies have shown that rs2278426 variation is associated with diabetes mellitus [26–28], dyslipidemia [29], metabolic syndrome [30], and coronary heart disease [28]. However, the involvement of this gene has rarely been reported in patients with pre-DM in China, particularly among the Han population in Hebei Province. In this study, T2DM and pre-DM patients of Han Chinese descent in the city of Shijiazhuang were selected as the research population. The association between rs2278426 and T2DM and pre-DM was examined by identifying polymorphisms of rs2278426.

## 2. Materials and Methods

### 2.1. Study Participants

We studied 1,444 people of Chinese Han descent from Hebei General Hospital, Shijiazhuang City, Hebei Province: 524 healthy controls, 460 patients with pre-DM, and 460 patients with T2DM. People with other types of diabetes, chronic kidney disease, or chronic liver disease were excluded. All participants received diet and exercise therapy but no medications that could affect blood glucose. Blood samples were collected from each participant at Hebei General Hospital from January 2015 to May 2016. All enrollees were given a 75 g oral glucose tolerance test, and blood glucose and insulin levels were measured 120 minutes later. We used the 1999 diagnostic criteria from the World Health Organization [31]: normal glucose tolerance was indicated by fasting plasma glucose (FPG) < 6.1 mmol/L and 2-hour postprandial glucose (2 h PG) < 7.8 mmol/L; prediabetes was indicated by impaired fasting glucose (IFT): 6.1 mmol/L ≤ FPG < 7.0 mmol/L and 2 h PG < 7.8 mmol/L; and impaired glucose tolerance (IGT) was indicated by FPG < 6.1 mmol/L and 7.8 ≤ 2 h PG < 11.1 mmol/L or IFT + IGT, with T2DM indicated by FPG ≥ 7.0 mmol/L or 2 h PG ≥ 11.1 mmol/L. Basic data included age, sex, body mass index (BMI), systolic blood pressure (SBP), and diastolic blood pressure (DBP). All participants were asked to fast for at least 10 hours before the collection of blood samples, and insulin, glucose-lowering drugs, and alcohol were stopped at least 12 hours beforehand. The research program was approved by the ethics committee of Hebei General Hospital. Participants voluntarily signed an informed consent form.

### 2.2. Biochemical Analysis

Blood samples were taken from all participants. FPG, total cholesterol (TC), triglycerides (TG), HDL, and LDL were measured by using a Hitachi 7600-110 automatic biochemistry instrument (Hitachi, Japan). Glycated hemoglobin was determined by ion exchange chromatography (ADAMS A1c HA-8180, Japan). Serum insulin was determined by electrochemical luminescence (ECL) (Roche cobas e 601 ECL instrument, Germany). Insulin resistance (IR) is a homeostasis model assessment (HOMA) indicator estimated by calculating *β*-cell steady state function (HOMA-B) and IR (HOMA-IR) using the HOMA2 calculator software version 2.2.3 (Oxford University Diabetes Trial Unit).

### 2.3. Blood Sampling and Genotyping

Genomic DNA of all the 1,444 samples was extracted from peripheral blood leukocytes using a genomic DNA kit (GENEray Biotechnology, Shanghai, China). We selected the single nucleotide polymorphism rs2278426 through the HapMap project and GenBand. The polymorphisms of *ANGPTL*8 were detected by the ligation detection reaction (LDR). The genotypes of the patients were determined using DNA sequencing on an ABI3730 genetic analyzer (Applied Biosystems, USA). The primers for amplification were designed with Primer Premier 5.0 software (Premier Biosoft Intl., CA, USA) as follows: 5'-CAGGAGTTCTATTGTGCGGC-3' (forward) and 5'-CCTGATGCAACTATCGCACC-3' (reverse); the melting temperature is 55°C. The PCR reaction system comprised the following: 7.5 *µ*L 2 × PCR reaction buffer, 2 *µ*L 10 *µ*mol/L mixed primer, 30 ng template DNA, and distilled H_2_O added to a total volume of 15 *µ*L. The real-time PCR cycling conditions were as follows: initial denaturing at 94°C for 3 min and 35 cycles of 94°C for 30 s (denaturing), 55°C for 15 s (annealing), and 72°C for 90 s (extension). For the LDRs, the probes (GENEray Biotechnology, Shanghai, China) were combined, mixed, and spun by centrifugation for 30 cycles after 30 seconds at 94°C and 3 minutes at 56°C. We used 1 *µ*L product, added 9 *µ*L highly deionized formamide, denatured the mixture at 95°C for 3 min followed by immediate transfer to an ice water bath, and then, sequenced the samples using GeneMarker V2.2.0 (PA, USA).

### 2.4. Statistical Analysis

Statistical procedures were carried out using SPSS software version 19 (IBM SPSS Statistics, Armonk, NY, USA). Continuous variables are expressed as means ± standard deviation, and normally distributed variables were analyzed by one-way analysis of variance. The Bonferroni test was further applied to the post hoc pairwise analysis to correct the *P* values. The Hardy–Weinberg equilibrium was applied to the *ANGPTL*8 genotype using Pearson's *χ*^2^ test among groups. The allele/genotype frequencies of the control, pre-DM, and T2DM groups were compared using a simple *χ*^2^ test, and the logistic regression technique was adjusted for age, sex, and BMI. All *P* values were two-tailed, and *P* values less than 0.05 were considered significant.

## 3. Results

### 3.1. Baseline Features

Population and medical data are shown in Table 1. Compared with the control group, BMI, SBP, and DBP were significantly higher in the pre-DM and T2DM groups. Additionally, FPG, 2 h PG, glycosylated hemoglobin (HbA1c), TC, and TG were all significantly higher in patients with pre-DM and T2DM. The level of HOMA-IR was significantly higher in the T2DM group, whereas the levels of HDL, fasting serum insulin, and HOMA-B were significantly lower in both the pre-DM and T2DM group patients than in the control group.

### 3.2. Genetic Association of the *ANGPTL*8 rs2278426 Polymorphism in All Groups

The genotype distribution of rs2278426 polymorphisms in the three groups was consistent with Hardy–Weinberg equilibrium. The TT genotype frequency was significantly higher in pre-DM patients than in healthy controls (6.8%, *P*=0.009). After binary logistic regression analysis adjusted for age, sex, and BMI, the odds ratio (OR) of the TT genotype in the pre-DM group was still higher than that of the control group (OR = 1.696, 95% confidence interval [CI] = 1.026–2.802, *P*=0.039). In T2DM patients, the frequency of the CT (48%) and TT (15%) genotypes were higher than those in the control group (CT: 40.1% and TT: 6.8%). After adjusting for age, sex, and BMI, the ORs were 1.384 for the CT genotype (95%CI = 1.013–1.890, *P*=0.041) and 2.530 for the TT genotype (95% CI = 1.476–4.334, *P*=0.001). Compared with the control group (26.9%), the frequency of the T allele was significantly higher in the pre-DM (32.8%) and T2DM (39%) groups (*P*=0.004; after adjusting for age, sex, and BMI, the ORs were 1.253 for the pre-DM group (95%CI = 1.017–1.544, *P*=0.034) and 1.518 for the T2DM group (95% CI = 1.214–1.897, *P*=0.001) (Tables 2 and 3). By analyzing the dominant, additive, genetic recessive, and co-dominant models of rs2278426, we found that the dominant, additive, and recessive models were significantly correlated with pre-DM (*P*=0.027, *P*=0.008, *P*=0.013, respectively; Table 2). In the T2DM group, all four models were significantly correlated with T2DM (*P*=0.001, *P*=0.001, *P*=0.001, *P*=0.012, respectively; Table 2).

The frequencies of the CT and TT genotypes in the pre-DM group were 43.9% and 10.9%, respectively, with corresponding OR values of 1.143 (*P*=0.359) and 1.696 (*P*=0.039), respectively.

The frequency of the T allele in the pre-DM group was significantly higher than that in the control group (OR, 1.253; *P*=0.034; Table 3). In the T2DM group, the frequencies of the CT and TT genotypes were significantly higher than those in the control group, with OR values of 1.384 (*P*=0.041) and 2.530 (*P*=0.001), respectively. Compared with the control group, both genotypes showed a significantly higher frequency of the T allele (OR, 1.518; *P*=0.001; Table 3).

### 3.3. Association of the *ANGPTL*8 rs2278426 Polymorphism with T2DM and Pre-DM Demographic and Biochemical Parameters

Biochemical parameters associated with *ANGPTL*8 rs2278426 in pre-DM and T2DM patients are listed in Tables 4 and 5. Compared with the CC and CT genotypes, pre-DM and T2DM patients with the TT genotype had significantly higher FPG, postprandial blood glucose, and Hb1Ac levels. In contrast, insulin levels in pre-DM and T2DM patients with the TT genotype were significantly lower than those with the CC and CT genotypes. We also found that the HOMA-B levels of patients with the CT (*P* < 0.001) and TT (*P* < 0.001) genotypes in the T2DM and pre-DM groups were significantly lower than those with the CC genotype. However, there was no significant association between *ANGPTL*8 rs2278426 polymorphism and other biochemical indicators.

## 4. Discussion

*ANGPTL*8 is a novel secretory protein that is composed of 198 amino acids, is mainly secreted by liver and adipose tissue, [32] and is closely related to glucose and lipid metabolism and insulin resistance [33, 34]. Related clinical studies have shown that serum *ANGPTL*8 is associated with diabetes and metabolic syndrome, [22, 35, 36] as well as chronic complications related to diabetes, including diabetic nephropathy and diabetic retinopathy [37, 38].

In this study, the TT genotype and T allele frequency at rs2278426 were significantly higher in the pre-DM group than in the control group. In the T2DM group, the frequencies of CT and TT genotypes and T alleles were significantly higher than in the control group. These results suggested that individuals with the T allele were more likely to develop pre-DM and T2DM, and this association persisted after adjustment for gender, age, and BMI. A study has shown that an Arab population of patients with non-type 2 diabetes with the rs2278426 CT genotype have significantly higher fasting glucose levels than CC genotype carriers [39]. Another study found that, in the Iranian population, the frequency of the CT gene at this locus was significantly higher in patients with T2DM than in the control group, suggesting that this genotype increased the risk of T2DM [26]. Additionally, a similar study of the rs2278426 polymorphism in the Japanese population found that a higher proportion of patients with T2DM and impaired glucose tolerance carried the CT or TT genotypes compared with the CC genotype [27]. The results of these studies are consistent with ours, all showing that gene polymorphism at *ANGPTL*8 rs2278426 leads to an increased risk of T2DM. Furthermore, the results of this study are consistent with those of the Japanese study with regard to the pre-DM population, which showed that polymorphism at this site also increases their risk of progression to T2DM. We also found that, compared with CC and CT genotype carriers, TT genotype carriers had higher levels of FPG, postprandial blood glucose, and Hb1Ac. While this was consistent with the studies mentioned above to a certain extent, we also found that the fasting insulin levels and HOMA-B were significantly lower in the TT genotype carriers compared with the CC and CT genotype carriers in pre-DM and T2DM. However, in studies by Ghasemi et al. [26] and others, insulin levels were significantly higher in CT compared with CC genotype carriers (*P*=0.005), but this difference was not found in TT genotype carriers. Additionally, a related study [40] in China found a trend of increasing fasting insulin level in CT + TT genotype carriers, but it was not significantly different than the CC genotype (*P*=0.427). We speculate that the reduced fasting insulin levels in TT genotype carriers may be caused by functional changes in *ANGPTL*8 that affect its role as a regulatory factor involved in the promotion of pancreatic *β*-cell proliferation. Further research is needed to confirm this possibility.

Similarly, the rs2278426 polymorphism of the *ANGPTL*8 gene might also be related to lipid metabolism. Among African American and Hispanic people, T allele carriers had lower levels of LDL-C than C allele carriers [17]. Similar results were observed in a study on the correlation between *ANGPTL*8 polymorphism and serum lipids in a healthy population in Guangxi [41]. However, there was no correlation between the single nucleotide polymorphism locus and serum lipid-related indexes in European American [17], Arab [39], and Han Chinese populations living in Jiangsu [40]. In our study, compared with the CC genotype, the LDL level of TT genotype carriers in the pre-DM group was significantly lower (*P*=0.024), and significant differences were found in other lipid indexes and with the T2DM group.

There are several explanations for the differences in results between our study and those of others. One is the MAF (minor allele frequency). In this study, the frequency of the minimum rs2278426 locus allele frequency in the control group was 6.8%, which is 5% higher than that of European Americans [17], close to the 6.18% for Arabs [39] and far less than the 18% for African Americans [17], 17.8% for Han Chinese in Guangxi [41], 19.5% for Han Chinese in Jiangsu [40], 23.04% for the Mulam ethnic group of Guangxi [41], and 26% for Spain [17]. Guo et al. [41] showed that there are racial differences in the allelic and genotypic frequencies of *ANGPTL*8 rs2278426 (C/T) and also in their relationship with other biochemical parameters. Furthermore, there is a linkage imbalance. One study has shown that rs2278426 (C/T) has a strong linkage with an rs34692794 mutation that changes the binding site of Pax4 [26]. Studies on Pax4, a conserved transcription factor, have shown that it is mainly expressed in the pancreas and plays an important role in promoting the hyposecretion of *β* cells in the islets of Langerhans [42–45]. Thus, impairment of this transcription factor can lead to insulin resistance and increased incidence of T2DM.

At present, the mechanism of the *ANGPTL*8 rs2278426 polymorphism is unclear. Some authors [46, 47] have speculated that the change in the amino acid sequence may affect the structure of the *ANGPTL*8 protein, damaging its functional domain and, thereby, affecting its function. However, the effect of this polymorphism on the function of *ANGPTL*8 requires further study.

This study is the first to show a significantly lower fasting insulin level in a group of pre-DM patients with the TT genotype compared with the CC genotype. In the T2DM group, fasting insulin levels in patients with the CT and TT genotypes were significantly lower than those with the CC genotype. This study is similar to our previous one [48]. This may be related to the study of the same population and the occurrence characteristics of diabetes in the Chinese population. Firstly, carbohydrate is the main functional substance, and secondly, insulin secretion and quality decrease [1]. The two genes which we studied are involved in the process of insulin secretion [16, 49], so we think they have association with these factors, and the specific mechanism needs to be supported by further research. As far as we know, this is also the first report on the association of the *ANGPTL*8 rs2278426 polymorphism with pre-DM, rather than T2DM, in a population of Han Chinese people.

Our study also has some limitations, the first being its cross-sectional design. Second, the relevant sample collection was mainly limited to the Shijiazhuang area of Hebei Province, although this was done intentionally to avoid regional influence. Third, we did not measure the levels of *ANGPTL*8 protein; thus, the effect of the rs2278426 polymorphism on the expression of *ANGPTL*8 and its potential mechanism in pre-DM and T2DM remain to be studied. Finally, we only studied one locus of *ANGPTL*8 (rs2278426). Because genetic polymorphism can also be regulated by genetic linkage imbalance, the study of additional loci might improve the results of future experiments.

## 5. Conclusions

The polymorphism of *ANGPTL*8 rs2278426 was significantly associated with pre-DM and T2DM and reduced fasting insulin levels. Additionally, individuals with the T allele had lower insulin levels than those with the C allele and may benefit from early insulin treatment once diagnosed with T2DM. Our findings should be replicated in a larger group of patients of different racial backgrounds to confirm these relationships.

## Figures and Tables

**Table 1:** Demographic and biochemical characteristics of participants in the healthy control, pre-DM, and T2DM patient groups.

Variables	Groups			All *P* values	Pairwise comparisons		
	Controls (*n* = 524)	Pre-DM (*n* = 460)	T2DM (*n* = 460)		P1	P2	P3
Age (years)	46.02 ± 13.46	53.64 ± 11.94	59.10 ± 11.10	<0.001	<0.001	<0.001	<0.001
Male/female (*n*)	199/325	200/260	208/252	—	—	—	—
BMI (kg/m^2^)	25.32 ± 3.87	27.10 ± 3.98	27.04 ± 3.76	<0.001	<0.001	<0.001	1.000
SBP (mm/Hg)	122.80 ± 12.64	126.03 ± 12.01	130.92 ± 12.52	<0.001	<0.001	<0.001	<0.001
DBP (mm/Hg)	74.68 ± 9.09	76.41 ± 8.72	77.95 ± 8.57	<0.001	0.007	<0.001	0.025
TC (mmol/L)	4.62 ± 0.91	4.94 ± 0.95	5.01 ± 1.03	<0.001	<0.001	<0.001	0.803
TG (mmol/L)	1.27 ± 0.76	1.62 ± 1.06	1.88 ± 1.50	<0.001	<0.001	<0.001	0.001
HDL (mmol/L)	1.41 ± 0.31	1.30 ± 0.24	1.30 ± 0.31	<0.001	<0.001	<0.001	1.000
LDL (mmol/L)	2.85 ± 1.44	3.04 ± 0.77	2.90 ± 0.82	0.019	0.017	1.000	0.171
FPG (mmol/L)	5.00 ± 0.33	5.74 ± 0.58	7.86 ± 1.83	<0.001	<0.001	<0.001	<0.001
PBG (mmol/L)	5.54 ± 1.03	8.20 ± 1.31	12.52 ± 2.76	<0.001	<0.001	<0.001	<0.001
FINS (*µ*U/mL)	9.66 ± 3.58	8.78 ± 3.11	7.82 ± 2.76	<0.001	<.001	<0.001	<0.001
Postprandial insulin (*µ*U/m)	42.18 ± 21.44	63.85 ± 22.32	47.08 ± 15.89	<0.001	<0.001	0.001	<0.001
HbA1c (%)	5.52 ± 0.31	5.77 ± 0.40	7.34 ± 1.52	<0.001	<0.001	<0.001	<0.001
HOMA-IR	2.15 ± 0.83	2.24 ± 0.84	2.72 ± 1.13	<0.001	0.343	<0.001	<0.001
HOMA-B	136.00 ± 64.31	84.13 ± 39.40	42.93 ± 24.56	<0.001	<0.001	<0.001	<0.001

Values are shown as mean ± standard deviation. Each group was compared by one-way analysis of variance. *P* values were corrected by a post hoc Bonferroni test. Pre-DM: prediabetes; T2DM: type 2 diabetes mellitus; BMI: body mass index; SBP: systolic blood pressure; DBP: diastolic blood pressure; TC: total cholesterol; TG: triglycerides; HDL: high-density lipoprotein; LDL: low-density lipoprotein; FPG: fasting plasma glucose; PBG: postprandial blood glucose; FINS: fasting insulin; HbA1c: glycosylated hemoglobin; HOMA-IR/B: homeostasis model assessment-insulin resistance/*β*-cell function. P1: pre-DM vs. controls; P2:T2DM vs. controls; P3:T2DM vs. pre-DM. Table 1 is reproduced from Hou et al. 2020 [48] (under the Creative Commons Attribution License).

**Table 2:** Genotype and allele frequencies for the *ANGPTL*8 rs2278426 (C/T) polymorphism in the study groups.

Genotypes rs659366	Healthy controls (*n* = 524)	Pre-DM (*n* = 460)	T2DM (*n* = 460)
CC	278 (53.1)	208 (45.2)	170 (37.0)
CT	210 (40.1)	202 (43.9)	221 (48.0)
TT	36 (6.8)	50 (10.9)	69 (15.0)
MAF (A-allele), %	26.2	32.9	39.0
P_HEW_	0.951	0.994	0.990
C	766 (73.1)	618 (67.2)	561 (61.0)
T	282 (26.9)	302 (32.8)	359 (39.0)
(CC + CT) vs. TT (dominant)	—	OR = 0.605 [0.386–0.947] *P*=0.027	OR = 0.418 [0.273–0.639] *P*=0.001
CC vs. TT (additive)	—	OR = 0.539 [0.339–0.857] *P*=0.008	OR = 0.319 [0.204–0.498] *P*=0.001
CC vs. (CT + TT) (recessive)	—	OR = 0.73 [0.568–0.939] *P*=0.014	OR = 0.519 [0.402–0.67] *P*=0.001
CT vs. (CC + TT) (co-dominant)	—	OR = 1.171 [0.908–1.509] *P*=0.224	OR = 1.383 [1.074–1.781] *P*=0.012

Values are shown as *n* (%). Pre-DM: prediabetes; T2DM: type 2 diabetes mellitus; MAF: minor allele frequency; CI: confidence interval; OR: odds ratio; HWE=Hardy–Weinberg equilibrium.

**Table 3:** Odds ratios of having each genotype and allele of the *ANGPTL*8 rs2278426 (C/T) polymorphism in the study groups.

Genotype/allele	Pre-DM vs. control				T2DM vs. control			
	P	OR	P^a^	OR	P	OR	P^a^	OR
rs2278426								
CC	—	Reference			—	Reference		
CT	0.062	1.286 (0.988–1.674)	0.359	1.143 (0.859–1.522)	0.001	1.721 (1.316–2.251)	0.041	1.384 (1.013–1.890)
TT	0.009	1.856 (1.167–2.954)	0.039	1.696 (1.026–2.802)	0.001	3.224 (2.057–5.052)	0.001	2.530 (1.476–4.334)
C	—	Reference			—	Reference		
T	0.004	1.327 (1.093–1.611)	0.034	1.253 (1.017–1.544)	0.001	1.751 (1.447–2.118)	0.001	1.518 (1.214–1.897)

OR: odds ratio; CI: confidence interval; pre-DM: prediabetes; T2DM: type 2 diabetes mellitus. P^a^ value adjusted for age, sex, and body mass index.

**Table 4:** Association of the *ANGPTL*8 rs2278426 (C/T) polymorphism genotypes with biochemical parameters in the pre-DM group.

Variables	Genotype groups			All *P* values	Pairwise comparisons		
	CC (*n* = 208)	CT (*n* = 202)	TT (*n* = 50)		P1	P2	P3
Age (years)	52.19 ± 12.75	54.82 ± 11.47	54.94 ± 9.70	0.06	0.077	0.428	1.000
Male/female (*n*)	94/114	89/113	17/33	—	—	—	—
BMI (kg/m^2^)	27.00 ± 3.76	27.36 ± 4.24	26.52 ± 3.78	0.362	1.000	1.000	0.548
SBP (mm/Hg)	125.94 ± 12.11	126.19 ± 11.93	125.80 ± 12.12	0.967	1.000	1.000	1.000
DBP (mm/Hg)	76.90 ± 8.57	76.17 ± 8.97	75.34 ± 8.31	0.46	1.000	0.771	1.000
TC (mmol/L)	4.93 ± 0.94	5.01 ± 0.96	4.72 ± 0.90	0.144	1.000	0.516	0.157
TG (mmol/L)	1.62 ± 1.05	1.67 ± 1.14	1.38 ± 0.75	0.213	1.000	0.426	0.237
HDL (mmol/L)	1.31 ± 0.24	1.29 ± 0.25	1.28 ± 0.24	0.692	1.000	1.000	1.000
LDL (mmol/L)	3.06 ± 0.77	3.08 ± 0.77	2.74 ± 0.72	0.017	1.000	0.024	0.018
FPG (mmol/L)	5.69 ± 0.62	5.73 ± 0.48	6.01 ± 0.71	<0.001	1.000	0.001	0.008
PBG (mmol/L)	7.93 ± 1.32	8.39 ± 1.10	8.56 ± 1.79	0.002	0.001	0.006	1.000
FINS (*µ*U/mL)	9.24 ± 3.20	8.59 ± 2.72	7.62 ± 3.81	<0.001	0.095	0.003	0.142
Postprandial insulin (*µ*U/m)	64.53 ± 23.46	64.70 ± 20.76	57.58 ± 22.96	0.109	1.000	0.144	0.131
HbA1c (%)	5.71 ± 0.36	5.79 ± 0.37	5.98 ± 0.57	<0.001	0.139	<0.001	0.007
HOMA-IR	2.34 ± 0.88	2.19 ± 0.73	2.05 ± 1.07	0.049	0.223	0.094	0.903
HOMA-B	92.50 ± 44.07	80.19 ± 31.02	65.25 ± 40.79	<0.001	0.004	<0.001	0.043

Values are shown as mean ± standard deviation. Each group was compared by one-way analysis of variance. *P* values were corrected by a post hoc Bonferroni test. Pre-DM: prediabetes; BMI: body mass index; SBP: systolic blood pressure; DBP: diastolic blood pressure; TC: total cholesterol; TG: triglycerides; HDL: high-density lipoprotein; LDL: low-density lipoprotein; FPG: fasting plasma glucose; PBG: postprandial blood glucose; FINS: fasting insulin; HbA1c: glycosylated hemoglobin; HOMA-IR/-B: homeostasis model assessment-insulin resistance/*β*-cell function. P1:CT vs. CC; P2:TT vs. CC; P3:TT vs. CT.

**Table 5:** Association of the *ANGPTL*8 rs2278426 (C/T) polymorphism genotypes with biochemical parameters in the T2DM group.

Variables	Genotype groups			All *P* values	Pairwise comparisons		
	CC (*n* = 170)	CT (*n* = 221)	TT (*n* = 69)		P1	P2	P3
Age (years)	58.35 ± 11.24	59.14 ± 11.23	60.84 ± 10.24	<0.291	1.000	0.350	0.800
Male/female (*n*)	71/99	106/115	31/38	—	—	—	—
BMI (kg/m^2^)	26.91 ± 3.71	27.19 ± 3.82	26.92 ± 3.73	0.745	1.000	1.000	1.000
SBP (mm/Hg)	131.20 ± 11.82	130.60 ± 13.08	131.26 ± 12.50	0.869	1.000	1.000	1.000
DBP (mm/Hg)	78.21 ± 8.97	77.71 ± 8.42	78.03 ± 8.09	0.848	1.000	1.000	1.000
TC (mmol/L)	4.96 ± 1.03	5.01 ± 0.98	5.14 ± 1.19	0.469	1.000	0.657	1.000
TG (mmol/L)	1.86 ± 1.44	1.88 ± 1.38	1.93 ± 1.98	0.950	1.000	1.000	1.000
HDL (mmol/L)	1.29 ± 0.32	1.31 ± 0.28	1.27 ± 0.28	0.505	1.000	1.000	0.825
LDL (mmol/L)	2.86 ± 0.829	2.91 ± 0.83	2.99 ± 0.83	0.526	1.000	0.785	1.000
FPG (mmol/L)	7.28 ± 1.66	7.84 ± 1.53	9.35 ± 2.29	<0.001	0.004	<0.001	<0.001
PBG (mmol/L)	11.86 ± 2.58	12.44 ± 2.64	14.44 ± 2.72	<0.001	0.095	<0.001	<0.001
FINS (*µ*U/mL)	8.17 ± 2.74	7.97 ± 2.72	6.44 ± 2.51	<0.001	1.000	<0.001	<0.001
Postprandial insulin (*µ*U/m)	47.33 ± 15.92	46.35 ± 15.56	48.80 ± 16.90	0.520	1.000	1.000	0.796
HbA1c (%)	7.08 ± 1.36	7.10 ± 1.21	8.75 ± 1.99	<0.001	1.000	<0.001	<0.001
HOMA-IR	2.64 ± 1.05	2.78 ± 1.11	2.72 ± 1.35	0.504	0.726	1.000	1.000
HOMA-B	51.49 ± 27.47	41.57 ± 21.04	26.25 ± 17.07	<0.001	<0.001	<0.001	<0.001

Values are shown as means ± standard deviation. Each group was compared by one-way analysis of variance. *P* values were corrected by a post hoc Bonferroni test. T2DM: type 2 diabetes mellitus; BMI: body mass index; SBP: systolic blood pressure; DBP: diastolic blood pressure; TC: total cholesterol; TG: triglycerides; HDL: high-density lipoprotein; LDL: low-density lipoprotein; FPG: fasting plasma glucose; PBG: postprandial blood glucose; FINS: fasting insulin; HOMA-IR: homeostasis model assessment-insulin resistance; HOMA-B: *β*-cell function. P1:CT vs. CC; P2:TT vs. CC; P3:TT vs. CT.

## Data Availability

The data that support the findings of this study are available from the corresponding author upon reasonable request.

## Abbreviations

*ANGPTL*8: Angiopoietin-like protein 8
BMI: Body mass index
DBP: Diastolic blood pressure
FINS: Fasting insulin
FPG: Fasting plasma glucose
HbA1c: Glycosylated hemoglobin
HDL: High-density lipoprotein
HOMA-IR/B: Homeostasis model assessment-insulin resistance/*β*-cell function
IFT: Impaired fasting glucose
IGT: Impaired glucose tolerance
LDL: Low-density lipoprotein
LPL: Lipoprotein lipase
MAF: Minor allele frequency
PBG: Postprandial blood glucose
Pre-DM: Prediabetes
SBP: Systolic blood pressure
T2DM: Type 2 diabetes mellitus
TC: Total cholesterol
TG: Triglycerides.
